# Integrative genomics and transcriptomics analysis of human embryonic and induced pluripotent stem cells

**DOI:** 10.1186/s13040-014-0032-2

**Published:** 2014-12-13

**Authors:** Kirsti Laurila, Reija Autio, Lingjia Kong, Elisa Närvä, Samer Hussein, Timo Otonkoski, Riitta Lahesmaa, Harri Lähdesmäki

**Affiliations:** Department of Information and Computer Science, Aalto University School of Science, Espoo, Finland; Department of Signal Processing, Tampere University of Technology, Tampere, Finland; School of Health Sciences, University of Tampere, Tampere, Finland; Turku Centre for Biotechnology, University of Turku and Åbo Akademi University, Turku, Finland; Samuel Lunenfeld Research Institute, Toronto, Canada; Research Program Unit, Molecular Neurology, Biomedicum Stem Cell Center, University of Helsinki, Helsinki, Finland

**Keywords:** hESC, hiPSC, Association analysis, SNP, CNV, Gene expression, Exon expression, Transcript expression

## Abstract

**Background:**

Human genomic variations, including single nucleotide polymorphisms (SNPs) and copy number variations (CNVs), are associated with several phenotypic traits varying from mild features to hereditary diseases. Several genome-wide studies have reported genomic variants that correlate with gene expression levels in various tissue and cell types.

**Results:**

We studied human embryonic stem cells (hESCs) and human induced pluripotent stem cells (hiPSCs) measuring the SNPs and CNVs with Affymetrix SNP 6 microarrays and expression values with Affymetrix Exon microarrays. We computed the linear relationships between SNPs and expression levels of exons, transcripts and genes, and the associations between gene CNVs and gene expression levels. Further, for a few of the resulted genes, the expression value was associated with both CNVs and SNPs. Our results revealed altogether 217 genes and 584 SNPs whose genomic alterations affect the transcriptome in the same cells. We analyzed the enriched pathways and gene ontologies within these groups of genes, and found out that the terms related to alternative splicing and development were enriched.

**Conclusions:**

Our results revealed that in the human pluripotent stem cells, the expression values of several genes, transcripts and exons were affected due to the genomic variation.

**Electronic supplementary material:**

The online version of this article (doi:10.1186/s13040-014-0032-2) contains supplementary material, which is available to authorized users.

## Background

After sequencing the human genome, numerous projects have focused on characterizing genomic alterations and associating them with the different diseases or functional elements of the genome. For example, the focus of two such projects, HapMap project [1] and 1000 genomes [2], is to identify the variants in the human genome. These variants are diverse and include *e.g.* single nucleotide polymorphisms (SNPs), insertions, deletions and copy number variations (CNVs) that comprise together 0.1% of the genome [3]. Moreover, the variants cause different types of phenotypic traits varying from mild properties such as eye color to severe hereditary diseases. These traits can be the consequences of alterations that are directly changing the protein function, or they can emerge after several gene expression regulation steps, caused for example by alternative splicing or methylation. Indeed, recent studies have shown that alterations in SNPs are of great importance as they can affect gene expression levels, alternative splicing, DNA methylation and miRNA-mediated gene expression levels in different types of cells [4-7]. Similarly, CNVs have been associated with changes in gene expression values in various cell types [8-10].

Several genome-wide studies have reported differences in *cis*-acting genomic variations between individuals and populations, in different cell types [11-14], and SNPs have also been associated with transcript isoform variation and alternative splicing [13-15]. Further, it has been reported that intronic SNPs are associated with both exon skipping events and complex traits, and that they are also predicted to result in protein domain changes [16]. Moreover, the correlation between SNPs and alternative splicing of exons is found to be the strongest at the exon-intron boundary, and the SNP closest to the alternative splicing event is most likely the functional one [4].

Genetic variants have been found to affect chromatin accessibility and transcription factor binding resulting in gene expression changes and phenotypic variation [17]. To that end, eQTLs are often (50%) DNase I sensitivity quantitative trait loci (dsQTLs) and majority of dsQTLs are located near genes [17]. Some of the *cis*-acting variations are similar across various cell types, while most of them can be detected in only some tissue and cell types [18]. These cell type specific variations differ between separate differentiation stages as has been shown with the cells of the hemapoietic system, using stem cells, progenitor cells, and differentiated cells of the myeloid and erythroid lineages [19]. On the other hand, when comparing human induced pluripotent stem cells (hiPSCs) with cells with less potency during the differentiation process, several autosomal allele-specific gene expressions remained similar during the differentiation process and were more dependent on genotypes than cell types even though more genes were expressed in hiPSCs [20]. However, similar behavior could not be detected in X chromosomal regions [20].

Despite the importance of the associations between the SNPs and other measurements, they have not yet been studied in human pluripotent stem cells. As most association studies are linking SNPs to a specific disease, there is no such known phenotype with the stem cells. The SNP arrays have been used in several studies of pluripotent stem cells and they have been mostly utilized for the copy number analysis [8,21-23]. By associating the SNPs to the gene expression levels we believe we can find new insights to the behavior of the pluripotent stem cells. As stem cells hold promise for the future medicine, the possible aberrations in the genome level with their associations to the transcriptomics must be recognized before the cells can be safely used for example in stem cell therapy [23].

In this study, we detected the effects of SNPs on expression values of both human embryonic stem cells (hESCs) and hiPSCs that are derived from fibroblasts. Further, we analyzed the associations between the gene copy numbers and gene expression values in hiPSCs. Similar associations between CNVs and gene expressions in hESCs have been reported earlier [8]. Previously, it has been shown that when different variants are associated with the gene expression levels, only a small part (<2%) of the SNPs (associated with 84% of gene expression differences) and CNVs (associated with 18%) overlap [24]. We studied the correlations between the SNPs and the expression levels of gene, transcript and exon expressions and the associations between the gene copy numbers and gene expressions. Further, we performed downstream analyses of the resulting *cis*-acting pairs and detected the overlapping expression changes associated with SNPs and CNVs.

## Methods

### Data sets

**Figure 1:**
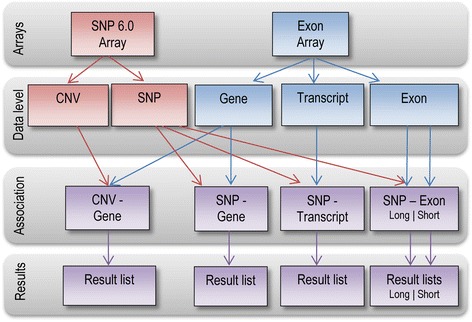
**The samples were measured with SNP arrays and exon arrays.** The associations are computed between the gene copy numbers and gene expression values. Further, the effects of the SNP changes on the transcriptomics data in the levels of genes, transcripts and exons with two intervals, long and short, are studied. The gene, transcript, and exon expression values, measured with the exon array, are illustrated in blue, while the CNV and SNP values obtained with the SNP 6.0 array are colored red. The arrows indicate the used values in each of the association analyses. The analysis results five lists of associations; 1) gene CNV – gene expression, 2) SNP – gene expression, 3) SNP – transcript expression, 4) SNP – exon (short) expression, and 5) SNP – exon (long) expression.

The analyzed data set consists of nine hESC, eight hiPSC and three fibroblast cell lines. The copy numbers and gene expression value alterations of the hESC samples (FES21,FES22, FES29, FES61, FES75, H9 (s14), H7 (s14) P38, H7 (s6) P132, H7 (s6) P237) were studied in [8] whereas the copy numbers of the hiPSC samples, reprogrammed from fibroblasts (FiPS1-14, FiPS2-10, FiPS2-13, FiPS3-12, FiPS5-3, FiPS5-7, FiPS6-3, FiPS6-12) and their parent fibroblast samples (IMR90, MRC5, HFF) were explored in [21]. Each of these 20 samples was hybridized to both Genome-Wide Human SNP 6.0 (Affymetrix) and GeneChip Human Exon 1.0 ST arrays (Affymetrix). The data can be downloaded from Gene Expression Omnibus (GEO) with series numbers GSE15097 (hESC data [8]) and GSE26173 (hiPSC and fibroblast SNP 6.0 array data [21]). The hiPSC and fibroblast expression data (GSE42625) are previously unpublished. The data analysis workflow can be seen in Figure 1.

### Exon array hybridizations

For measuring the expression values of the hiPSC samples, the RNA was isolated using RNeasy Kit (Qiagen) and DNase I (Qiagen) digestion was performed to eliminate DNA from RNA samples. The concentration of the samples was measured with Nanodrop and the expression values of all hESC, hiPSC and fibroblast samples were measured with the Affymetrix GeneChip Human Exon 1.0 ST arrays. All the samples were hybridized in the Finnish Microarray and Sequencing Centre (Turku, Finland) according to manufacturer's protocol as described in [8] and [21].

### Effect of gene copy number on expression values

The CNVs of the genes were detected with Affymetrix Genotyping Console (3.0.2) utilizing the Birdseed v2 algorithm and were analyzed against the 40 in house hybridized HapMap samples (available in GSE15097) as reported in [21]. We used the regional GC correction, and the variations with at least five markers and length 10 kb in hiPSCs [21] and 50 kb in hESCs [8] were considered to have a CNV. All the variations were linked to Ensembl genes (build 49, corresponds the genome version hg18). The gene expression values of the exon array samples were computed with the *aroma.affymetrix* [25] package of Bioconductor [26] in R [27]. The probe values were directly linked to Ensembl genes (build 49) with the CDF files provided by *aroma.affymetrix* and preprocessed with the RMA method [28]. We performed the integration analysis for all the genes having a duplication or deletion in copy number in at least one sample. All the gene values in each sample were labeled based on the copy number value as gain, normal or loss. In the integration analysis, for each gene we computed a weight value ${w}_i=\frac{\left({m}_{G1}-{m}_{G0}\ \right)}{\left(st{d}_{G1}+st{d}_{G0}\right)}$ where the *m*_G1_ is the mean expression value and *std*_G1_ is the standard deviation of the gene *i* in those samples where the gene *i* was detected to be gained, and *m*_G0_ and *std*_G0_ are the mean and standard deviation of the expression values of the gene in those samples where the copy number of the gene has not altered [8,9,29-31]. The weight value corresponds to the difference between the groups. The weight value is high in cases where the distance between the mean values of the groups is large and the deviations within the groups are small. Therefore, high weight value for duplication indicates that gene expression is likely to be over expressed due to duplication. Similarly, we computed the weight values for the genes with the loss in their CNV. We computed the p-values for each weight value by 10000 permutations, by permuting the sample labels based on the permutation events. Further, the p-values were adjusted with the Benjamini-Hochberg method.

### Effect of SNPs on expression values

**Figure 2:**
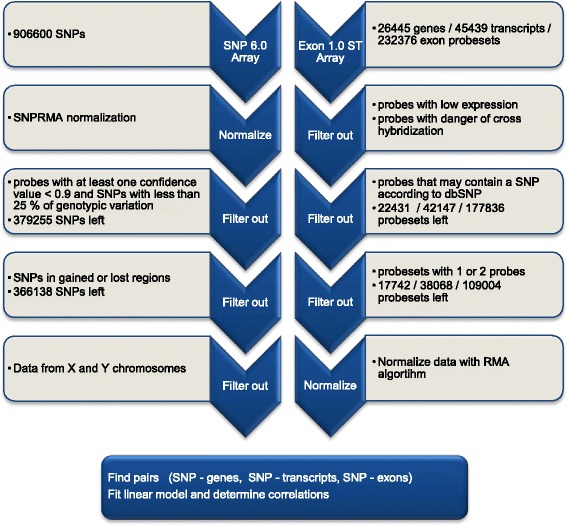
**The scheme of the SNP-expression analysis steps.** The description of each step includes also the numbers of the remaining gene/transcript/exon probesets after the filtering. The numbers for the SNP array are for the data set with all the samples. In the analysis of a single group of cells (hESCs or hiPSCs) the number of SNPs after the second filtering step is slightly lower due to the lack of the genotypic variation.

The analysis between the SNPs and expression values was performed using R [27] and Bioconductor [26] packages and the study was run in three different groups: for hESCs, hiPSCs and for all samples (hESCs, hiPSCs and fibroblasts) together. The analysis steps and the numbers of analyzed events after each analysis step are described in Figure 2.

Exon array data were analyzed separately in the levels of exons, transcripts and genes using custom CDF files, version 11 [32] linking the probes to Ensembl (build 49, corresponds the genome version hg18) genes, transcripts and exons. Additionally, several probes providing possibly unreliable information were filtered out before the actual correlation analysis. In the first filtering step, probes with low expression were considered to be background probes and thus filtered out. The limit for the background probes in Human Exon 1.0 ST Array (26 445 gene / 45 439 transcript / 23 2376 exon probesets) was separately defined for every G + C content using the antigenomic probes of the exon array. This limit was the average plus two standard deviations of the intensity of the antigenomic probes, and for each genomic probe of the array the maximum probe intensity across all the samples was compared with the limit to qualify the probe between the background and non-background probes [13]. In addition to the filtering of the low intensity probes, the probes that could be cross-hybridized were filtered out [33]. Moreover, since single nucleotide polymorphisms (SNPs) at probe sequences can affect the intensities of probes [34-37], especially they can severely bias exon expression estimates in individuals, not when using pooled samples [38], we filtered out the probes whose sequences had an SNP (SNP locations were determined using dbSNP version 129 [39]). As some of the exons are small, this QC filtering can result in removing some SNP-exon pairs, in which an SNP is located inside the exon and could contain a real association signal. Nevertheless, this additional QC step mainly removes false positives. Finally, the probesets with only one or two probes were masked out to avoid the unreliability of the expression values [32]. This filtering dramatically reduced the number of analyzed probesets. For example in the gene level analysis, the original 26 445 different probesets are reduced to 17 742 due to the filtering and in the exon level analysis the effect is even larger when more than half of the probesets are filtered out (Figure 2). After the filtering, the expression values were computed with the Robust Multi-array Average (RMA)-normalization method [40] of the Bioconductor's affy –package [41].

SNP 6.0 arrays were analyzed using the Bioconductor *oligo* package [42]. First, the array data was normalized using the SNPRMA normalization and the genotypes for each of the 906 600 SNPs of the array were determined with the Correct Robust Linear Model with Maximum likelihood based distance (CRLMM) method [42]. Further, the SNPs with less than 25% of genetic variation among the samples, were filtered out as well as the probes with a confidence value smaller than 0.9 in at least one of the samples. Remarkably, the number of filtered SNPs varied between the analyzed groups (hiPSCs, hESCs and all samples) as the proportion of the genetic variation differs in them. In the cases where an SNP was filtered out from the analysis for the whole data set (*i.e.* 20 samples) it was removed of the hiPSCs or hESCs groups also. Finally, we filtered out the SNPs occurring in the regions reported to have copy number changes in hESCs [8] and in hiPSCs [21]. Likewise, we excluded the SNPs within regions of the gained chromosomes of the samples having a mosaic karyotype.

After the data preparation for the SNPs and expressions, the SNPs were linked to exons, transcripts and genes. For the exons the linking was performed with two different ways; the SNP was considered to be linked to the studied exon if the polymorphism occurred 1) in the exon sequence or in the adjacent intron regions (short interval) or 2) in the whole gene area (long interval). An SNP located within the transcript region was considered to be linked to a transcript, as well as to a gene if located within or 5000 bps up- or downstream of the gene region. The linear relationships were detected with correlation analysis separately performed for each SNP - exon / transcript / gene pair by fitting a linear model for data (homozygous genotypes had values 1 and 3 and the heterozygous one had the value 2) by linear regression analysis with R's *lm* function. The correlation p-values were adjusted with Benjamini-Hochberg multiple testing correction method.

For validation, we studied the overlap between our associations and associations in rSNPBase [43]. We studied also the relation of the expression correlating SNPs with transcription factors. Specifically, the possible binding of the key embryonic stem cell transcription factors (*NANOG, OCT4, SOX2, E2F4*) to the SNP-regions was explored. This was performed by searching the overlapping SNP locations and the parts of the reported transcription factor binding sites (TFBSs) for the key stem cell factors identified with chromatin immunoprecipitation microarray (ChIP-chip) [44]. We further studied possible overlap between TFBSs and the SNPs by predicting the transcription factor (TF) binding to the SNP regions. The prediction of TF binding was computed using the TRANSFAC (Release 2010.2) [45] human binding motif position weight matrices (PWMs) (altogether 618 models) with the pseudocount 0.005. The binding was scored for the sequences of both SNP alleles by sliding the PWM over the flanking SNP sequence and computing the maximum binding score using uniform background probabilities [46]. Only the sequences with at least 80% of the maximum possible binding score on either of the alleles were further studied by computing the absolute difference of the binding scores between the alleles. We also studied if the genes, whose expressions were correlated with SNPs, showed enrichments in different pathways, networks or annotations. These analyses were performed with core analysis of Ingenuity Pathway Analysis (IPA) with Benjamini-Hochberg multiple testing correction method and with the Database for Annotation, Visualization and Integrated Discovery system (DAVID) [47,48].

## Results and discussion

### Copy number variation association with gene expression in hiPSCs

**Figure 3:**
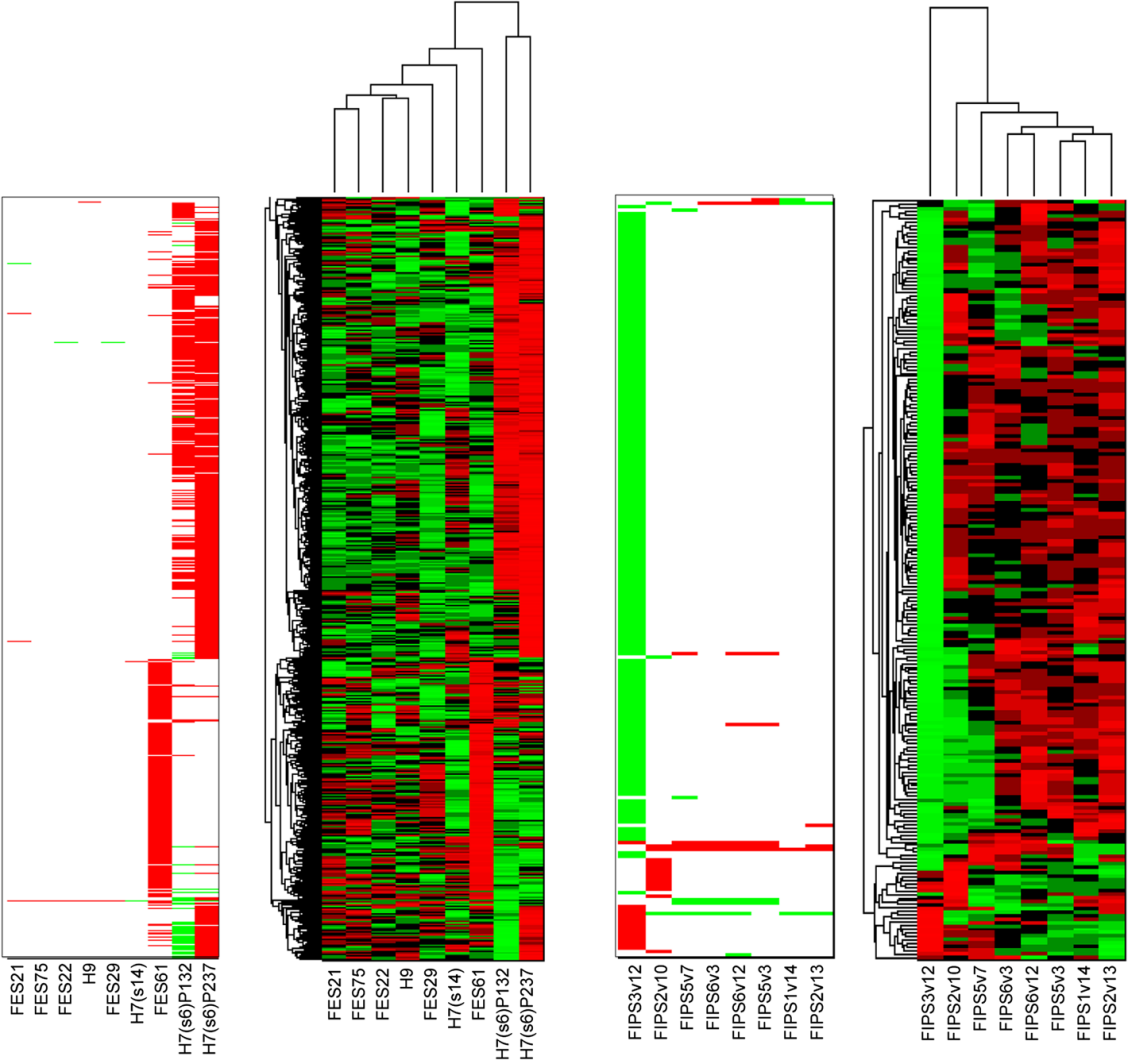
**Expression and copy number values of the genes having a copy number variation in at least one of the hESC (on the left) and hiPSC samples (on the right) across all the samples in the analysis.** The copy numbers are labeled with green - loss, white - no change, red -gain. The expression values are normalized row-wise, and thus the color of each gene indicates the value of the gene in respect to the other samples, red denoting high expression and green low expression value.

In the analysis of hiPSC samples, 139 genes were detected to have a gain and 359 genes a loss within the gene area in at least one of the samples (Additional file 1: Table S1). With adjusted p-value <0.05 we detected together 29 genes having significant association between a gain in copy number and a high expression value, and 188 genes between a loss in copy number and a low expression value (Table 1, Figure 3, Additional file 1: Table S1). For these resulting genes, the average logarithmic fold change between the expression values of the copied and normal samples is 0.589 (fold change 1.50) and the average logarithmic fold change between the expression values of the normal and lost samples 0.568 (fold change 1.48). The results of a similar association analysis of gene copy numbers and the expression values of hESCs were reported in [8]. The hESC data included larger regions with copy number changes when compared to the hiPCS data. As copy number detection was performed for hESC and hiPSC separately, the parameters were chosen separately for different data sets to optimize CNV detection. Therefore, tighter cut-offs for calling CNVs in the copy number analysis were used for the hESC samples. In the hESC samples altogether 6248 genes were detected to be gained, of which 1866 genes had a significant association between the CNV and expression. Further, 220 genes in hESC samples were detected to have a loss at least in one sample, of which 90 genes were significantly associated with the low expression value [8] (Figure 3). Further, 32 of these genes were detected to have association with both loss and low expression and gain and high expression value.

**Table 1:** **Top genes with highest FC change between the average gene expression values between the gained and normal and lost and normal samples**

**HGNC**	**Gene**	**Gene description**	**Logarithmic fold change**	**Number**	**Adjusted p-value**
GSTT1	ENSG00000184674	Glutathione S-transferase theta-1	1.8701	1	<0.0139
KCNMA1	ENSG00000156113	Calcium-activated potassium channel	1.7972	1	<0.0139
RNFT1	ENSG00000189050	RING finger and transmembrane	1.0443	1	<0.0139
APPBP2	ENSG00000062725	Amyloid protein-binding protein	0.9715	1	<0.0139
SMCHD1	ENSG00000101596	Structural maintenance of chromosomes exible hinge domain-containing protein 1	0.85418	1	<0.0139
NDC80	ENSG00000080986	Kinetochore protein NDC80 homolog	0.80433	1	<0.0139
HEATR6	ENSG00000068097	HEAT repeat-containing protein	0.77267	1	<0.0139
HLA-DRB5	ENSG00000198502	HLA class II histocompatibility antigen, DRB5 beta chain precursor	0.72371	5	<0.0139
METTL4	ENSG00000101574	Methyltransferase-like protein	0.70292	1	<0.0139
PPM1D	ENSG00000170836	Protein phosphatase 1D	0.6493	1	<0.0139
PDPN	ENSG00000162493	Podoplanin precursor (Glycoprotein 36)	-2.9885	1	<0.036
ALPL	ENSG00000162551	Alkaline phosphatase, tissue nonspecific isozyme precursor	-2.16	1	<0.036
NPPB	ENSG00000120937	Natriuretic peptides B precursor	-1.6065	1	<0.036
RCAN3	ENSG00000117602	Calcipressin-3 (Regulator of calcineurin 3)	-1.2506	1	<0.036
DFFA	ENSG00000160049	DNA fragmentation factor subunit alpha	-1.1259	1	<0.036
TPRG1L	ENSG00000158109	Tumor protein p63-regulated gene 1-like protein	-1.1232	1	<0.036
CDA	ENSG00000158825	Cytidine deaminase	-1.0503	1	<0.036
DDI2	ENSG00000197312	Protein DDI1 homolog 2	-1.045	1	<0.036
MAD2L2	ENSG00000116670	Mitotic spindle assembly checkpoint protein MAD2B	-1.0315	1	<0.036
AOF2	ENSG00000004487	Lysine-specific histone demethylase 1	-1.018	1	<0.036
ATAD3B	ENSG00000160072	ATPase family AAA	-1.0031	1	<0.036

The p-values are computed for the weight value with the permutation test and adjusted with the Benjamini-Hochberg criteria.

**Figure 4:**
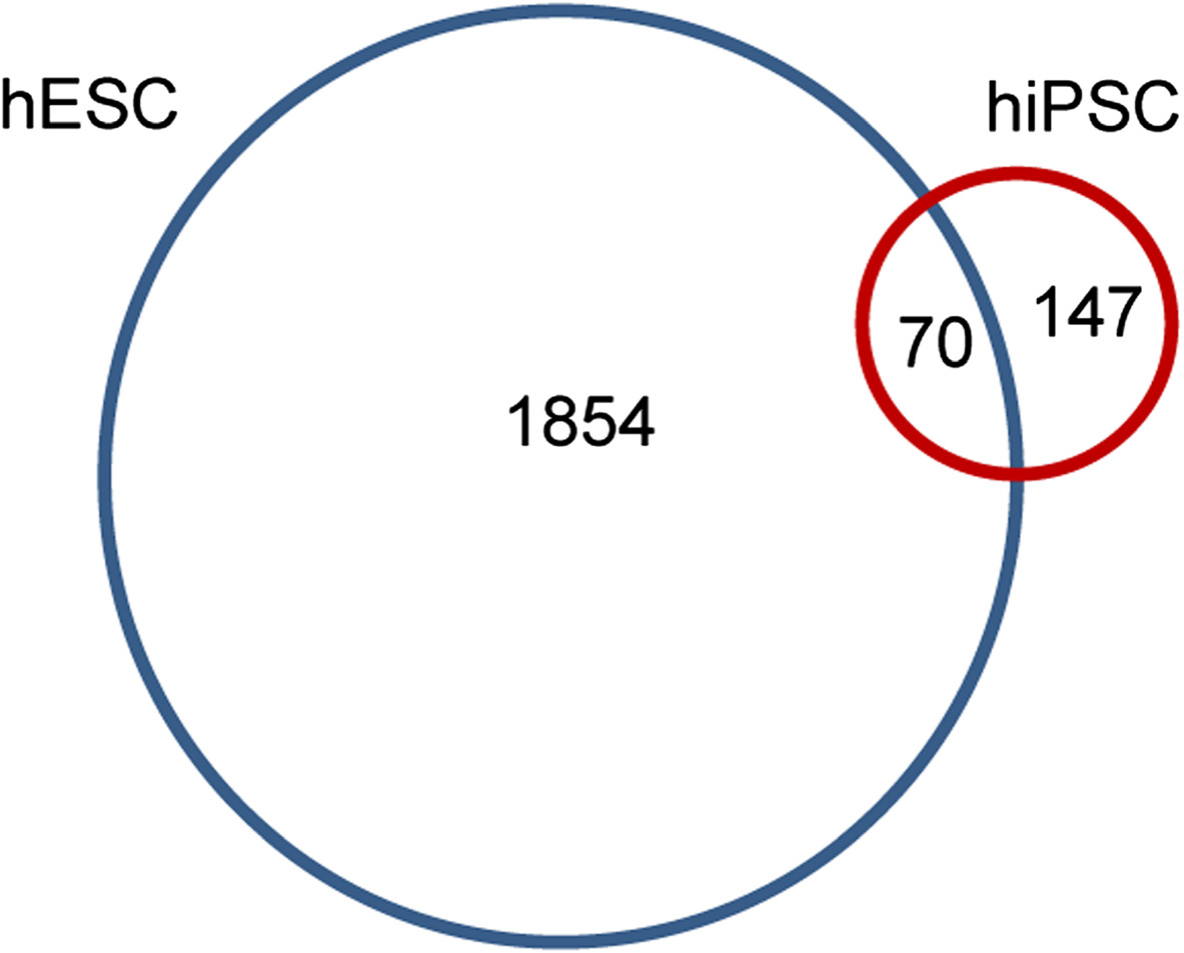
**Venn-diagram of the genes detected to have significant association between the copy number and gene expression in hESCs and hiPSCs.**

Based on our results, altogether 70 genes have a significant association between the copy number and expression value in both hiPSC and hESC samples (Figure 4). There is a clear difference in the number of genes whose copy number has altered in the hiPSCs and hESCs. In the hESC data, the long passage H7 samples are included, in which large parts of the chromosomes have been duplicated [8]. In the hiPSC samples there are no such huge CNVs, which is further the reason for the smaller number of detected genes. The largest variations in the hiPSC data are in chromosome 1 of the sample FiPS3-12 and chromosome 9 in FiPS1-14 (Additional file 2: Figure S1).

For the most of these 217 genes having association in hiPSC, the copy number of the gene is altered only in one sample. Especially the clear majority of the genes that are detected to have an association between the loss and low expression are locating in 1p36 which is only lost in the sample FiPS3-12 (Additional file 1: Table S1, Additional file 2: Figure S1). This deletion does not occur in the parental fibroblast sample. However, there are many genes whose copy numbers have altered in several samples, such as *SUMF1* (ENSG00000144455, Sulfatase-modifying factor 1 precursor) and *CFHR1* (ENSG00000080910, Complement factor H related protein 1 precursor) that are lost in the fibroblast sample HFF and in all the four hiPSC samples derived from HFF, and also further significantly associated with the low expression value in hiPSCs. In addition, genes *TBC1D3F* (ENSG00000189309, protein coding TBC1 domain family member 3B) and *TBC1D3C* (ENSG00000205019) gained in the IMR fibroblast sample are copied not only in the hiPSCs derived from IMR but also in hiPSCs derived from other fibroblasts, and are further having a significant association between the gained copy number and a high expression value in hiPSC samples. The gene *HLA-DRB5*, (ENSG00000198502, HLA class II histocompatibility antigen) which is gained in the fibroblast samples HFF and MRC5, is also gained in all the hiPSCs derived from HFF and MRC5, and has further an association between the gain and the expression value. Interestingly, the gene *DEFB4* (ENSG00000171711, Beta-defensin 2 precursor) is gained in three hiPSC samples and is significantly associated to a high expression value even though the copy number in all fibroblast samples is normal. In addition, there are two genes *SLC30A6* (ENSG00000152683, protein coding Zinc transporter 6) and *TUBA8* (ENSG00000183785, protein coding Tubulin alpha-8 chain) that are gained in all of the fibroblast and hiPSC samples. In total, 4% of the genes detected with an association between the changed copy number and expression value had a copy number variation already in the fibroblast samples (*i.e.* 5/29 of the gained associations and 3/188 of the lost associations), while 96% of these associated genes had a normal copy number value in fibroblasts. Interestingly, our analysis detected genes for which the CNV in their region is actually associating negatively with the expression value. For the gene CALB1, the deletion in copy number data resulted with more than 1.5 fold higher gene expression value, as well as gains have decreased with 1.5 fold the expression for NLGN4Y, TMPRSS11, NEBL, GPC6 and C10orf113 (Additional file 1: Table S1). However, these negative correlations were not confirmed with good p-values.

Furthermore, the functionality of the genes with positive association between the copy number and the expression value in the hiPSC samples was detected with the enrichment analysis on gene ontologies and pathways. Regulation of RAS protein signal transduction, organelle localization, macromolecule catabolism, as well as alternative splicing, alcaloid, coenzyme and secondary metabolism terms are significantly enriched (Additional file 3: Table S2).

### SNP association with exon, transcript and gene expression in hiPSCs and hESC

We computed the correlations between SNP genotypes and a gene/transcript/exon expression value by fitting a linear regression model between the genotype and expression values in hiPSCs, hESCs and combined group of hiPSCs, hESCs, and fibroblasts. As previous studies have reported some problems in microarray measurements, for example hybridization caused by SNPs in the probe areas, the cross hybridization of samples to several probes, and the uncertainty in the genotype determination [34-37], we filtered out several SNPs and exon array probes to reduce the number of false detections from the association analysis. After the filtering, the number of fitted models varied from 30 000 to almost 1.5 million in comparison types (gene/transcript/exon) between the groups (hiPSCs, hESCs, all), (see Methods). We fitted a linear regression model for each SNP-expression pair and estimated the significance of the models with the adjusted p-values of the slope of the model. For each comparison, the numbers and characteristics of the correlating pairs with adjusted p-value < 0.10 are listed at Table 2 (the full lists of correlating pairs in each comparison in Additional file 4: Table S3).

**Table 2:** **Results of different SNP correlation analyses**

**Expression measurement**	**Area of SNPs**	**Group**	**Number of significant correlation pairs/number of all pairs**	**Number of unique genes/transcripts/exons affected**	**Number of unique genes**	**Number of correlating SNPs inside/outside gene/transcript/exon regions**
Gene	gene5000bp	hiPSC	0/100363	-	-	-/-
Gene	gene5000bp	hESC	206/129416	56	56	190/16
Gene	gene5000bp	All	27/149986	20	20	22/5
Transcript	Transcript	hiPSC	5/197884	5	1	5/-
Transcript	Transcript	hESC	1/231846	1	1	1/-
Transcript	Transcript	All	22/296333	20	8	22/-
Exon	Short	hiPSC	0/75180	-	-	-/-
Exon	Short	hESC	0/95038	-	-	-/-
Exon	Short	All	53/113050	42	35	4/49
Exon	Long	hiPSC	0/821655	-	-	-/-
Exon	Long	hESC	0/955091	-	-	-/-
Exon	Long	All	651/1230955	342	159	4/647

Short SNP area means the area of the studied exon and adjacent introns, long area means the whole gene area.

**Figure 5:**
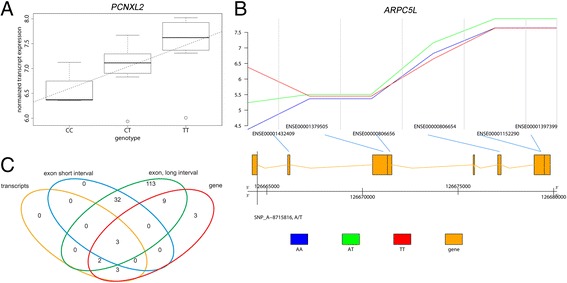
**Examples of the genes having correlation between genotypes and expression values and Venn-diagram of the results of the SNP-analysis. A)** Linear regression between the SNP_A-2052714 genotypes and gene *PCNXL2* (ENSG00000135749, pecanex-like 2) expression. The dashed line illustrates the computed linear regression line. The expression data for each genotype is visualized using the boxplot representation. **B)** The gene structure of *ARPC5L* (ENSG00000136950, actin related protein 2/3 complex, subunit 5-like), the location of exon ENSE00001432409 expression correlating SNP SNP_A-8715816 (rs10986468) and the mean expressions of samples of each genotype. The normalized logarithmic expression values (y-axis) and the exon with the genomic location (x-axis) are connected with light blue line. Some of the exons are not represented in exon array and thus their expression values are not measured. **C)** Summary of the genes in each SNP analysis. Venn-diagram illustrates the detected genes in the levels of genes, transcripts and exons with two intervals.

When comparing hiPSCs and hESCs separately, only a few or none of the correlating pairs could be identified, whereas while fitting the model to the whole data set, numerous correlating pairs were detected. We believe that this is due to the low number of samples and a conservative multiple testing correction method. As a result, we detected genes with a correlation between the SNP and expression value, such as *PHLDB2* (ENSG00000144824, pleckstrin homology-like domain, family B, member 2), affected by the genotypic variation in SNP_A-4263698 (dbSNP code rs698360, alleles C/T), which has lower gene expression in the samples with the genotype CC than in the samples with the genotype TT while the heterozygous CT genotype is related to a medium expression value of the gene (Figure 5A). Further, the SNP_A-8715816 (dbSNP code rs10986468, alleles A/T) has an effect on the expression of the second exon of the *ARPC5L* gene, within which it is also located (Figure 5B). While the other probesets of this gene are measuring steady expressions of the exons of this gene, with the genotype AA in SNP_A-8715816 the expression is clearly down-regulated.

Within the resulted SNPs correlating with expression values, often only two genotypes existed among the samples. This indicates that heterozygosity in a single nucleotide can have significant effect on the expression levels of genes. The detected variants in genotypes were equally common among different cell types, and thus these findings seem to be independent of the stem cell type. Further, as the fibroblasts were included in this part of the analysis, the results indicate that differentiated cells can also have similar effects. Based on our analysis of separate groups of hiPSC and hESC data, only few SNPs correlate with transcript expression and none with exon expression. However, in our analysis the sample size in the individual cell types is small, and as at least 25% of variation in an SNP is required, some of the interesting correlations might have been filtered out. Nevertheless, our results indicate that the expression differences in stem cells can be caused by SNPs and therefore they should be taken into account when considering the differential expression and alternative splicing.

Most of the associations in the levels of genes, transcripts and exons, were detected when all the samples were combined into one sample group (Table 2). Our study revealed three SNPs having an effect on the expression values at all the levels of genes, transcripts and exons (Figure 5C). Further as the datasets overlap, if the transcript expression is affected, the effect can always be seen at the gene level too. The majority of the effects in the exon levels do not occur at the gene levels, indicating potential cases of exon skipping or other alternative splicing events. Our results showed also that the affecting SNPs are located usually at the introns or at one of the other exons when affecting the exon expression, and within the gene region (not in the up-/downstream regions), when affecting the gene expression. Also, we did not detect any SNP enriched locations. The locations of SNPs detected to be associated with the expression values of genes, transcripts and exons are illustrated with POMO [49] in Additional file 5: Figure S2.

In total we found 584 SNPs that were associated with the expression value. Of these SNPs 438 (75%) have been reported in other eQTL studies and 458 (78%) of them were involved in RNA binding protein mediated regulation based on the rSNPBase [50]. Further, we overlapped the detected SNP – expression associations in the gene level to the list of rSNP related genes in the rSNPBase, and found out that 88% of our SNP- gene pairs and 95% of the SNP – transcript pairs were found also in rSNPBase. As expected, almost all of our SNP – exon pairs were not detected in the rSNPBase because rSNPBase had only associations in gene level, and most our exon level findings were exon specific and did not correspond to whole gene association in our data either. Further, when we compared the SNP locations with the stem cells key transcription factors (TF) (*NANOG, OCT4, SOX2, E2F4*) binding regions according to the ChIP-chip measurements [44], none of the SNPs are located in the binding sites. Next, we studied if the SNPs could affect other TF binding sites by comparing the binding scores computed according to TRANSFAC position weight matrices, and detected 29 unique transcription factors with a large difference in the binding score (Additional file 6: Table S4). Thus, this analysis suggests transcriptional regulators, which may have a causal role in regulating these genes in stem cells.

The functional analysis using the protein information resource (PIR) [50] for the exons, transcripts, and genes associating with an SNP genotype variation (SNP located in gene region) showed enrichment for alternative splicing and splice variant terms. Further, the Ingenuity Pathway Analysis (IPA, Ingenuity® Systems, www.ingenuity.com) showed that in several networks our result genes are not the most strategic genes of the network, but rather the targets of other network molecules instead (Additional file 6: Table S5, Table S6). For example in the network "Cellular Development, Embryonic Development, Organ Development", one third of affected molecules (*ATXN1, C9orf3, KCNJ3, NUAK1*) are indirect targets of *TGFB1*. Similar effects could be seen in other networks as well. In particular, several of these networks are related to the development, such as embryonic development, cardiovascular system development, tissue development *etc.* (Additional file 6: Table S6). This is however understandable, as 85% of the samples in the analysis are pluripotent stem cells.

### Overlap between SNP and CNV association results

Further, we wanted to know if the associated expression pairs with SNPs are the same ones as the expressions associated with CNVs. Therefore, we compared the genes in each group and found that four genes could be found in both of the results, all these genes are detected in hESC samples. Genes *ARNTL2* (ENSG00000029153, aryl hydrocarbon receptor nuclear translocator-like 2) and *PPP1CC* (ENSG00000186298, protein phosphatase 1, catalytic subunit, gamma isozym) are both in gained regions and genes *CDS2* (ENSG00000101290, CDP-diacylglycerol synthase) and *LRRN4* (ENSG00000125872, leucine rich repeat neuronal 4) are in loss regions. Thus for these four genes, we cannot rule out the possibility that the gene expression differences might be related actually to copy number variations, and not to the genotype. All the other associations occurred only either between SNPs and the expression value or between CNV and expression value, which indicates that most likely they were not due to other genomic variation. This small amount of overlapping genes in these analyses also confirms the finding that the CNV and SNP associations with expression levels overlap only slightly [24].

## Conclusions

In this paper, we have analyzed human embryonic and induced pluripotent stem cells with two different methods for finding associations between genomic variations *i.e.* SNPs and CNVs, and the transcriptomics data. The results revealed several associations between gene copy numbers and the gene expression values, and also some associations between the SNPs and exon, transcript and gene expression values. Several copy number associations can be found in hiPSCs suggesting similar features with genomic instability as has been described in hESCs [8]. In hiPSCs data, we detected altogether 217 genes, *i.e.* ~1% of all genes, of which copy number variation were associated with the expression value. Further, after careful filtering the suspicious SNP probes, we had 366138 SNPs of which 584 (0.16%) were significantly associated with the expression value in at least one of the analysis done for the hESCs, hiPSCs and combined group of samples. When also transcript and exon results were studied in the gene level, we had together 721 associations between SNP and genes.

Some integrative analyses have already been performed where individual variation has been linked to transcription factor binding [51,52] or to DNA methylation [53]. We believe that these type of analyses need to be correlated with the functional parameters of differentiated stem cells in order to understand how the genetic and epigenetic variability of pluripotent stem cells translates into the performance and safety of their differentiated progeny. Although we report several such genotype-transcriptome effects here and as earlier results have shown [22], further studies are needed to understand the significance of such associations. Meanwhile the size of the sample groups in such studies should be enlarged to detect the subgroups having certain association. Particularly in cell therapy studies, it would be essential to have the detailed information of such associations.

## Additional files

Additional file 1: Table S1. The associations between the gene copy number and the gene expression values. The sheet 1 includes the copy numbers and gene expression values of all the genes gained in the hiPSC samples. The column *Mean Label Gain* is the mean of the logarithmic expression values of the gene in samples that have a gain in the copy number, and *Mean Label No Gain* in samples that do not have a gain. The weight and p-values are from the association test, and the adjusted p-value shows the adjusted p-value. The sheet 2 gives the same information for the lost genes. Additional file 2: Figure S1. Copy number changes of the hiPSC samples a) FiPS3-12 and b) FiPS1-14. The blue regions represent gains and the red regions losses. Additional file 3: Table S2. The results of the enrichment analysis of the genes that are associated between the copy number and the gene expression value. Analysis was computed with the DAVID software and EASE score. Additional file 4: Table S3. The results of the correlation analyses between the SNPs and the expressions levels. The exons, transcripts and genes in hESCs, hiPSCs and in the combined group of hESCs, hiPSCs and fibroblasts are correlated with SNPs and the relationships are validated using SNP-Gene associations of rSNPBase. Additional file 5: Figure S2. Illustration of the locations of the SNPs detected to have association with expression values. Associations found in hESCs are marked as green, in hiPSCs as blue and in the combined group of hESCs, hiPSCs and fibroblasts as red. The outermost ring indicates the cytobands, and the other rings from outer to inner are SNPs associated with genes, transcripts, exons with short interval and exons with long interval. Additional file 6: Table S4. Changes in transcription factor binding scores. BSD = binding score difference, max BS = maximum possible binding score, (h) = hESC, (l) = long interval for SNPs, (s) = short interval for SNPs. **Table S5.** Top functions of IPA analysis in the SNP genotype correlated genes. **Table S6.** Top networks of IPA analysis in the SNP genotype correlated genes. The genes that are associated with SNPs in the analysis are bolded.
