# Supplementary figures and images for: Integrative genomics and transcriptomics analysis of human embryonic and induced pluripotent stem cells
Source: BioData Min. 2014 Dec 13;7:32. doi: 10.1186/s13040-014-0032-2 (PMC4298950; doi:10.1186/s13040-014-0032-2)

A)

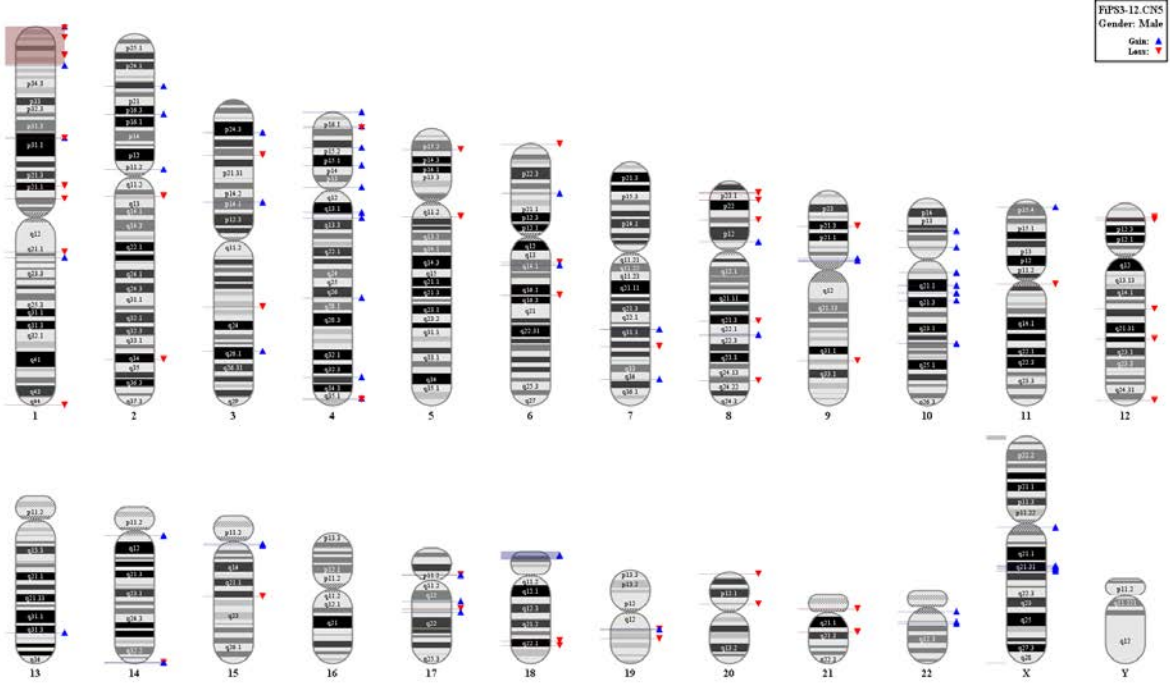

B)

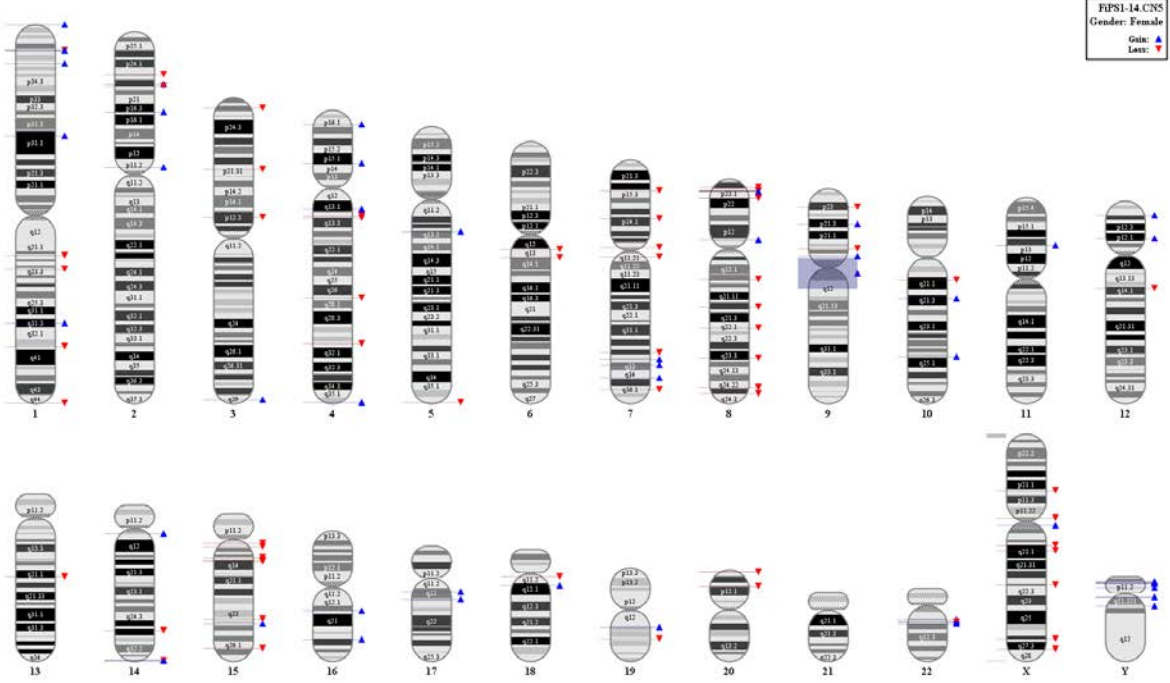

Supplement: Additional file 2: Figure S1. — Copy number changes of the hiPSC samples a) FiPS3-12 and b) FiPS1-14. The blue regions represent gains and the red regions losses. [file 13040_2014_32_MOESM2_ESM.pdf]

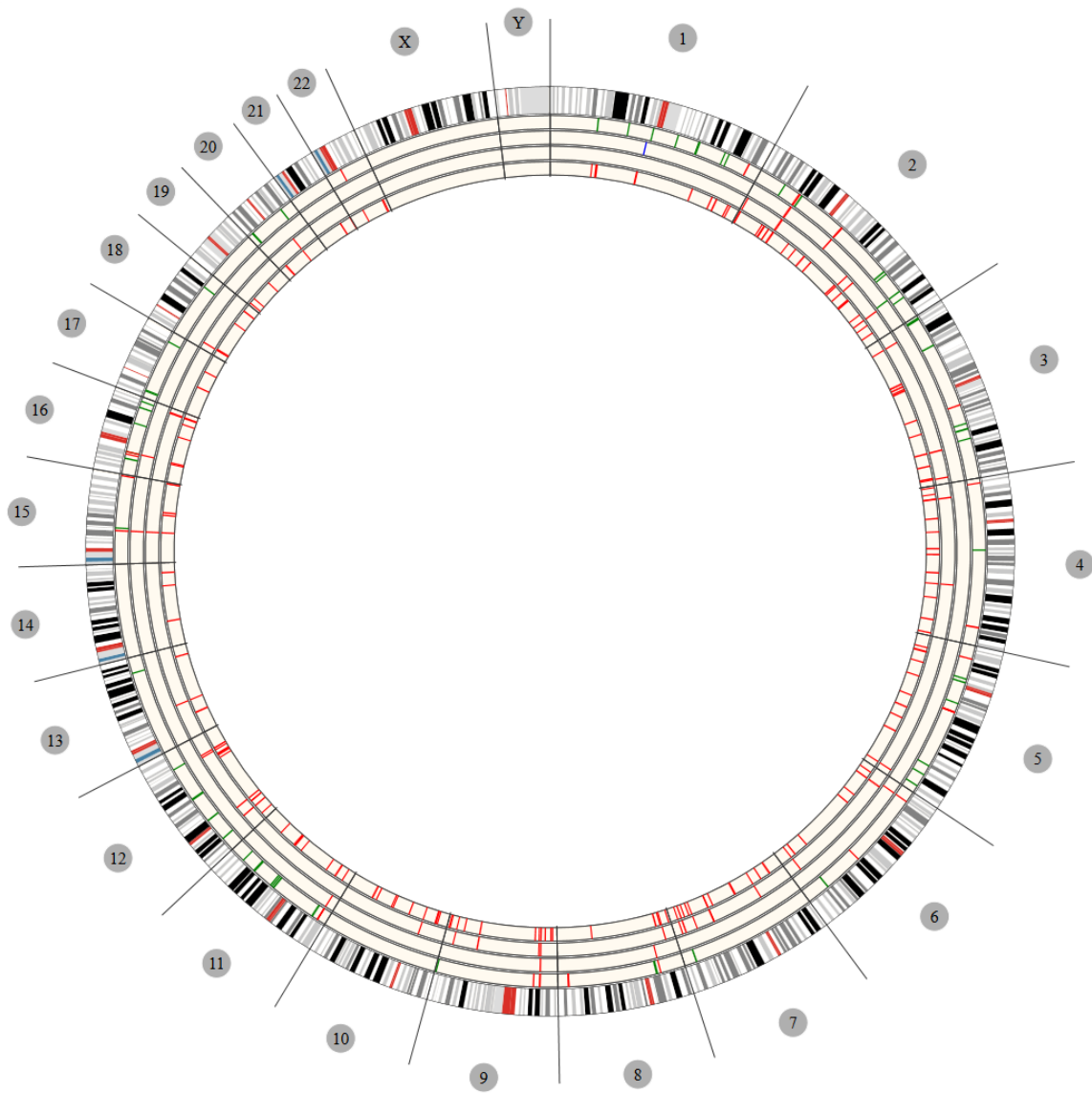

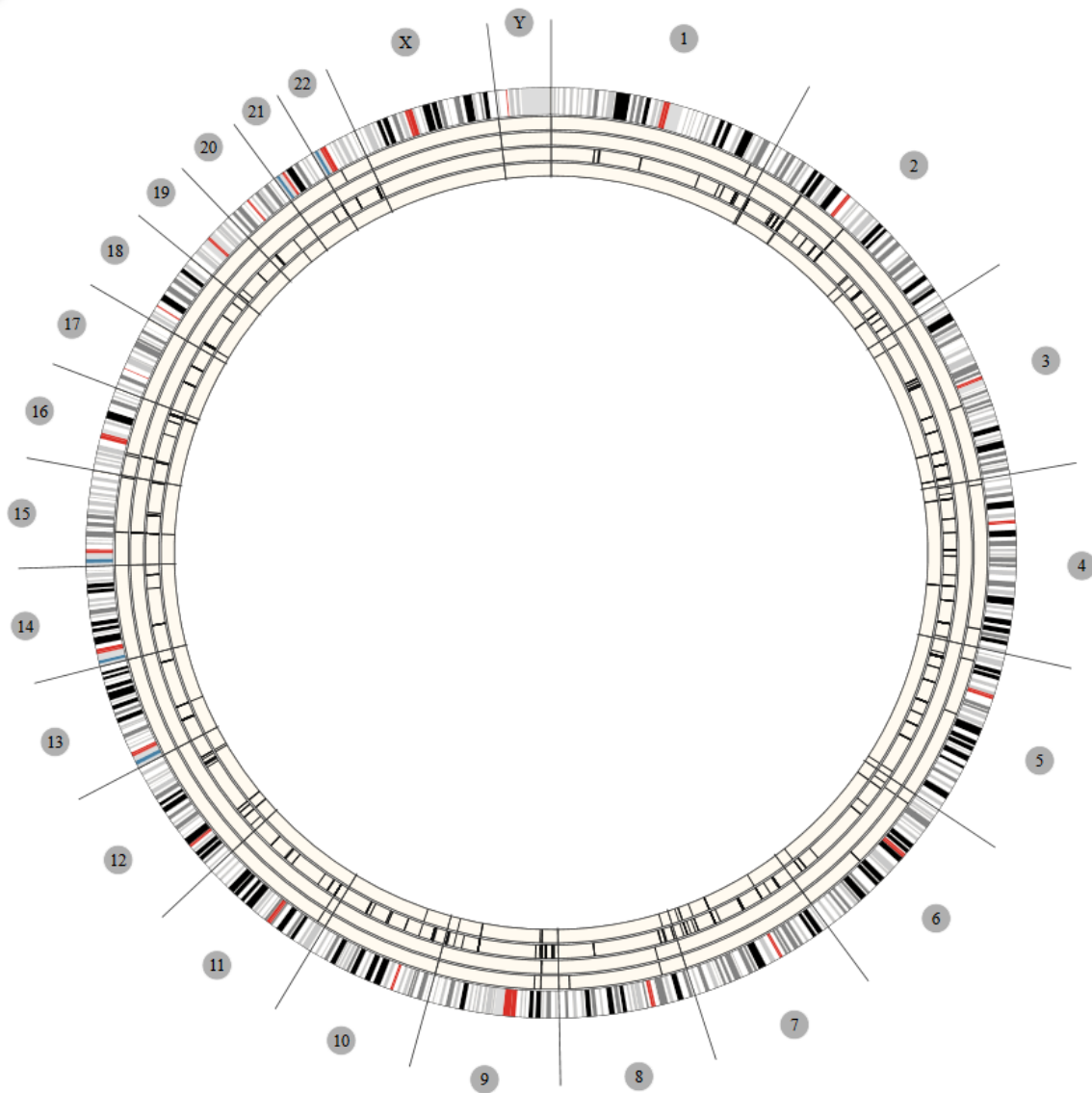

Supplement: Additional file 5: Figure S2. — Illustration of the locations of the SNPs detected to have association with expression values. Associations found in hESCs are marked as green, in hiPSCs as blue and in the combined group of hESCs, hiPSCs and fibroblasts as red. The outermost ring indicates the cytobands, and the other rings from outer to inner are SNPs associated with genes, transcripts, exons with short interval and exons with long interval. [file 13040_2014_32_MOESM5_ESM.pdf]
