# Supplementary material for: Integrative genomics and transcriptomics analysis of human embryonic and induced pluripotent stem cells
Source: BioData Min. 2014 Dec 13;7:32. doi: 10.1186/s13040-014-0032-2 (PMC4298950; doi:10.1186/s13040-014-0032-2)
Supplement: Additional file 6: Table S4. — Changes in transcription factor binding scores. BSD = binding score difference, max BS = maximum possible binding score, (h) = hESC, (l) = long interval for SNPs, (s) = short interval for SNPs. Table S5. Top functions of IPA analysis in the SNP genotype correlated genes. Table S6. Top networks of IPA analysis in the SNP genotype correlated genes. The genes that are associated with SNPs in the analysis are bolded. [file 13040_2014_32_MOESM6_ESM.pdf]

Supplementary table 4: Changes in transcription factor binding scores. BSD=binding score difference, max BS= maximum possible binding score, (h)=hESC, (l)=long interval for SNPs, (s)=short interval for SNPs

SNP	alleles	Ensembl code	gene	association	Transfac TF	score of allele A	score of allele B	BSD	max BS
SNP_A-8646384	C/T	ENSG00000144668	ITGA9	gene(h)	V_CREBP1CJUN_01 (ATF2/c-Jun)	21947	109	21838	21947
SNP_A-1830368	A/G	ENSE00001169516	APBB2	exon (l)	V_LMO2COM_02 (Lmo2complex)	4784	52	4732	5251
SNP_A-1988240	A/G	ENSE00000765449	TFB1M	exon (l)	V_GATA1_06 (GATA-1)	33160	177	32983	33160
SNP_A-4288522	A/G	ENSG00000144668	ITGA9	gene (h)	V_HEB_Q6 (HEB)	21095	174	20921	21095
SNP_A-1939907	A/C	ENSG00000158258	CLSTN2	gene (h)	V_OSF2_Q6 (Osf2)	11297	56	11241	11297
SNP_A-2303118	A/G	ENSE00001443673	TFB1M	exon (l)	V_OSF2_Q6 (Osf2)	11297	1961	9337	11297
SNP_A-8453065	A/T	ENSE00001021726	CDH18	exon (l)	V_HMGIY_Q6 (HMGIY)	14	1849	-1835	2214
SNP_A-8512399	G/T	ENSG00000158258	CLSTN2	gene (h)	V_HMGIY_Q6 (HMGIY)	2214	12	2202	2214
SNP_A-8613122	A/C	ENSG00000149972	CNTN5	gene (h)	V_HMGIY_Q6 (HMGIY)	1849	9	1839	2214
SNP_A-1830368	A/G	ENSE00001169516	APBB2	exon (l)	V_GATA_Q6 (GATA)	1485	250	1235	1485
SNP_A-1988240	A/G	ENSE00000765449	TFB1M	exon (l)	V_GATA_Q6 (GATA)	1485	59	1426	1485
SNP_A-2218269	C/G	ENSE00001008804<br>ENSE00001146532	ACOXL<br>ACOXL	exon (l)<br>exon(l)	V_MYC_Q2 (Myc)	3995	20	3975	3995
SNP_A-2296358	C/T	ENSE00001217678	AFF3	exon (l)	V_MYC_Q2 (Myc)	3995	20	3975	3995
SNP_A-8406487	C/G	ENSG00000119537	KDSR	gene (h)	V_CREB_Q3 (CREB)	793	62	731	891
SNP_A-8618328	A/C	ENSG00000175928	LRRN1	gene (h)	V_LEF1_Q2 (LEF1)	2069	10	2059	2069
SNP_A-4288522	A/G	ENSG00000144668	ITGA9	gene (h)	V_AP4_Q6_01 (AP-4)	9233	64	9169	9233
SNP_A-8672281	A/T	ENSE00000780527<br>ENSE00000968103<br>ENSE00001291680<br>ENSE00001298842<br>ENSE00000780525<br>ENSE00000780526	FNDC3B<br>FNDC3B<br>FNDC3B<br>FNDC3B<br>FNDC3B<br>FNDC3B	exon (l)<br>exon (l)<br>exon (l)<br>exon (l)<br>exon (l)<br>exon (l)	V_HNF4_Q6_02 (HNF4)	498	3	495	498
SNP_A-4283012	A/G	ENSG00000174332	GLIS1	gene (h)	V_HOXA7_01 (HOXA7)	4949	25	4924	5552
SNP_A-2218269	C/G	ENSE00001008804<br>ENSE00001146532	ACOXL<br>ACOXL	exon (l)<br>exon (l)	V_CLOCKBMAL_Q6 (CLOCK:BMAL)	14698	73	14625	14698
SNP_A-2165062	C/T	ENSG00000171631	P2RY6	gene (h)	V_KID3_01 (Kid3)	360	2	358	360
SNP_A-4279956	C/T	ENSE00001436130<br>ENSE00001435333	PCNXL2<br>PCNLX2	exon (l)<br>exon (l)	V_KID3_01 (Kid3)	360	2	358	360
SNP_A-4282973	C/T	ENSE00001021726	CDH18	exon (l)	V_KID3_01(Kid3)	360	2	358	360
SNP_A-8299044	C/T	ENSG00000158258	CLSTN2	gene (h)	V_KID3_01(Kid3)	360	2	358	360
SNP_A-8303622	C/T	ENSE00001157163	KCNAB1	exon (l)	V_KID3_01 (Kid3)	360	2	358	360
SNP_A-8361400	C/T	ENSG00000164318	EGFLAM	gene (h)	V_KID3_01 (Kid3)	360	2	358	360
SNP_A-8362388	C/G	ENSE00001220387<br>ENSE00001220387	LEPREL1<br>LEPREL1	exon (l)<br>exon (s)	V_KID3_01 (Kid3)	360	2	358	360
SNP_A-8365058	C/T	ENSE00001531459	COL6A5	exon (l)	V_KID3_01 (Kid3)	360	2	358	360
SNP_A-8402354	C/T	ENSG00000131781	FMO5	gene (h)	V_KID3_01 (Kid3)	360	2	358	360
SNP_A-8677910	C/T	ENSE00001157163<br>ENSE00001157163	KCNAB1<br>KCNAB1	exon (l)<br>exon (s)	V_KID3_01 (Kid3)	360	2	358	360
SNP_A-8692395	C/G	ENSE00001497139	LAMA1	exon (l)	V_KID3_01 (Kid3)	360	2	358	360
SNP_A-8552484	A/G	ENSE00001124309	UST	exon (l)	V_PARP_Q3 (PARP)	8668	43	8625	8668
SNP_A-2129437	A/G	ENSE00001455839<br>ENSE00001030729	NRD1<br>NRD1	exon (l)<br>exon (l)	V_ZNF333_01 (ZNF333)	951	5	946	951
SNP_A-8591611	A/G	ENSG00000144331	ZNF385B	gene (h)	V_ZNF333_01 (ZNF333)	951	5	946	951
SNP_A-1921773	A/G	ENSG00000174332	GLIS1	gene (h)	V_ELF1_Q5 (ELF1)	2322	12	2310	2322
SNP_A-2044682	A/G	ENSG00000205629	LCMT1	gene (h)	V_ELF1_Q5 (ELF1)	2322	12	2310	2322
SNP_A-2182766	G/T	ENSE00001479582	NRCAM	exon (l)	V_ELF1_Q5 (ELF16)	2322	15	2307	2322
SNP_A-2265109	A/G	ENSE00000874857	WDR59	exon (l)	V_ELF1_Q5 (ELF1)	2322	12	2310	2322
SNP_A-8497366	A/C	ENSG00000158258	CLSTN2	gene (h)	V_ELF1_Q5 (ELF1)	2322	12	2310	2322
SNP_A-2088972	C/T	ENSE00001436130<br>ENSE00001435333	PCNXL2<br>PCNXL2_16	exon (l)<br>exon (l)	V_IPF1_Q6 (IPF1)	1046	201	845	1046
SNP_A-8705838	C/T	ENSE00000759874	MAN2A1	exon (l)	V_IPF1_Q6 (IPF1)	1046	201	845	1046
SNP_A-1884247	A/G	ENSE00000739600<br>ENSE00000820317	NRCAM<br>NRCAM	exon (l)<br>exon (l)	V_NFAT1_Q6 (NFAT1)	1764	65	1699	1764
SNP_A-1884431	A/G	ENSE00001001657	DNAJC15	exon (l)	V_NFAT1_Q6 (NFAT1)	1764	65	1699	1764
SNP_A-8552261	A/G	ENSE00001380455	UNC79	exon (l)	V_NFAT1_Q6 (NFAT1)	1764	65	1699	1764
SNP_A-8613122	A/C	ENSG00000149972	CNTN5	gene (h)	V_NFAT1_Q6 (NFAT1)	1764	9	1756	1764

Supplementary table 5: Top functions of IPA analysis in the SNP genotype correlated genes.

Group	IPA function	Number of genes
Gene	Cellular assembly and organization	4
	Cell death	4
	Cell-to-Cell Signaling and Interaction	5
Gene (hESC)	Cell signaling	3
	Carbohydrate metabolism	8
	Cell cycle	7
	Cellular development	8
	Molecular transport	7
Transcript	Cellular function and maintenance	3
Exon (short)	Cellular development	6
	Cellular growth and proliferation	7
	Cellular movement	6
	Cell-to-Cell Signaling and Interaction	6
	Cell cycle	4
Exon (long)	Cell morphology	23
	Cellular assembly and organization	28
	Cellular movement	24
	Cell cycle	7
	Cellular growth and proliferation	25

Supplementary table 6: Top networks of IPA analysis in the SNP genotype correlated genes. The genes that are associated with SNPs in the analysis are bolded.

Group	Top functions of network	Molecules of network	# mol
Exon long	Cellular Movement Endocrine System Disorders, Immunological Disease	Actin,Alpha Actinin,**ARHGAP22**,**ARPC2**,Cadherin,**CDH13**,**CDH18**,**DLC1**,**DOT1L**,ERK1/2,F Actin,FSH,G protein beta gamma,**GATA4**,hCG,**HIPK3**,Histone h3,**KCNJ3**,**KDM4C**,**LPP**,**MBD1**,**NRXN1**,**PALLD**,**PLCB1**,Pld,**PRKCB**,**PTPRM**,**RALGDS**,Ras homolog,**RASGRP3**,**SORBS2**,**SSH1**,**STK17A**,**TTN**,**WDR1**	24
	Connective Tissue Disorders, Genetic Disorder, Dermatological Diseases and Conditions	Alp,**ANKH**,**CDKN2B**,**COL13A1**,**COL18A1**,**COL21A1**,**COL25A1**,**COL5A1**,**COL6A5**,collagen,Collagen type I,Collagen type IV,Collagen(s),**CUX1**,Cyclin A,Cyclin D,Cyclin E,**ESRRG**,**FBLN2**,**FGF2**,**HDAC9**,HISTONE,**IGFBP7**,**ITGA1**,**LAMA1**,**LAMB1**,Laminin,Laminin1,MEF2,,NFkB (complex),**NID1**,PDGF BB,**SLBP**,Tgf beta,**TNC**	20
	Gene Expression, Infection Mechanism, Cancer	AR,beta-estradiol,C11orf10,C9orf5,CHCHD8,CPA2,**CPS1**,**DENND5A**,**ELMOD1**,FAM53C,,FOS,GINS3,HNF4A,**LDB2**,MLH1,NGFR,**NR2C1**,NR3C1,**NRD1**,**PHLDB2**,**POLR3E**,**PSD3**,RAE1,RBM6,**RCL1**,SLC38A1,**TFB1M**,**TLN2**,TMEM87B,TNFSF11,**TRIM37**,**TTC38**,**USP30**,**WDR59**,YWHAZ	16
	Endocrine System Disorders, Genetic Disorder, Metabolic Disease	AJAP1,**APBB2**,**ARHGAP24**,**ARPC5L**,ARRB2,ATP2C1,BCL9L,**C1QTNF3**,CA4,Ca2+,CDH8,CNTN3,CTNNB1,DLG4,**DQX1**,**FAM40A**,**FBN2**,**FOXP1**,**FRAS1**,HRAS,HTT,**KCNAB1**,KLF16,**L3MBTL3**,**PCCA**,**PTPRG**,**QPCT**,RCN1,**RGL1**,RSF1,SCYL2,SFXN3,**SLC24A2**,TBR1,trimethylamine	16
	Cancer, Cardiovascular System Development and Function, Hematological Disease	ADRA1D,ANGPTL2,**ANKS1B**,beta-estradiol,**BICD1**,**CCDC141**,CRNKL1,DISC1,**DOK6**,ERBB2,ETFB,FEZ2,**FMN1**,FNBP1,**FRMD4A**,GPR64,GPR88,GPR139,GPR173,GPR137B,HSD17B12,**KIAA1409**,PRR15L,**PXDN**,**RCL1**,RPL17,**SNTG2**,**SPAG1**,**SPOCK2**,**SYNM**,TRAF3IP1,VEGFA,YWHAQ,ZNF197,**ZNF398**	14
	Cell Cycle, Gene Expression, Cancer	**AEBP2**,**ARHGEF10**,ARHGEF17,**ARSB**,**AUTS2**,CDKN2A,**CECR1**,CLEC2A,**CPA4**,DEFA4,**HHAT**,IFNG,LGMN,**MAN2A1**,MAN2B1,MDN1,**MEIS2**,MORC2,Myb,MYC,**NAV2**,OMG,**PLEKHB1**,RARRES1,RBBP7,RHOA,Rpl9 (includes others),RT1-B,SCPEP1,tretinoin,**TRIP12**,VSIG4,Wnt,WNT2B,WNT9A	12
	Gene Expression, Cardiovascular System Development and Function, Cell Morphology	14-3-3,**ACACA**,Akt,**ATP8A2**,**ATXN1**,**BMP6**,C1orf182,Ck2,**CNGB1**,**DAB1**,Dnajb1-Hsp70,ERK,Focal adhesion kinase,**FRMD4B**,Hsp70,HSPA7,**HSPA14**,HSPA12A,Insulin,Jnk,**MAGI1**,Mapk,**NRCAM**,P38 MAPK,PGLYRP1,PI3K (complex),Pkc(s),PLC gamma,**PPFIBP1**,Ras,SRC,**TIAM1**,TRIAP1,Vegf,Vegfr dimer	12
	Cell Cycle, Respiratory System Development and Function, Carbohydrate Metabolism	AASS,ALDH5A1,Anti-inflammatory Cytokine,**ARNTL2**,**C9orf3**,CAK,CCL23,CCND1,CFI,CHST2,CHST3,CUL7,**CUL9**,**DLG2**,FASLG,**FNDC3B**,GSS,**IFT74**,IL13,IL17RB,**MPP4**,**MYOF**,NDST1,**NFIB**,ONECUT1,PDGF-CC,PI4K2B,PLXNC1,PMEPA1,RBX1,**TAPT1**,TDO2,TGFB1,**UST**,WNT5B	11
	Cell-To-Cell Signaling and Interaction, Cardiovascular System Development and Function, Cell Morphology	ATP,CDC42,**CRIM1**,DNAJB3,DNAJB13,DNAJC2,**DNAJC12**,DNAJC15,DNAJC16,DNAJC18,DNAJC22,DNAJC24,DNAJC25,DNAJC27,DNAJC28,DNAJC30,DNAJC5B,DNAJC5G,**EMID2**,**FANCC**,**FMNL2**,HSP,HSPA7,HSPA12A,HSPA12B,HSPB11,MYCN,**MYO1E**,**NUAK1**,**POLR1A**,**TBC1D9**,TBP,TNF,**VASH1**,**WDR62**	10
Exon short	Cancer, Cardiovascular System Development and Function, Tissue Development	Actin,ANTXR2,**ATXN1**,BCAM,CAP2,**CPA4**,DOCK3,ESR1,**FBLN2**,**FGF2**,FOLH1,FSH,GAL3ST1,**GATA4**,GSTZ1,ITGB4,ITGB8,**LAMA1**,**LAMB1**,Laminin,Laminin2,LRG1,LYPD3,MYO16,**NRXN1**,PPP6R2,Rac/Cdc42,REG3A,SERPINF,SERPINF2,**SSH1**,**STK17A**,TINAG,TLR7,**ZNF398**	11
	Cell Cycle, Cellular Development, Cellular Growth and Proliferation	Acot5,AQP8,BUD31,C1QB,**C9orf3**,Ca2+,CDHR1,**CDKN2B**,**CPS1**,**FMN1**,FNBP1,GIN1,HNF4A,JUN,KCNG1,**KCNJ3**,LPCAT3,mir-143,MRTO4,NOC3L,**NRCAM**,**NUAK1**,PPARA,**PXDN**,RAB18,SBNO2,SHISA5,SKI,**SLC24A2**,SLC35A1,SLC35A5,SLC39A1,**TFB1M**,TGFB1,TTC22	10
	Cellular Movement, Cellular Development, Hematological System Development and Function	**AEBP2**,AIF1,**ARPC5L**,ARRB2,BAZ1A,beta-estradiol,**BICD1**,BOP1/LOC727967,CDC42EP4,**CDH13**,CTPS,GSK3B,IL20,**KCNAB1**,KCND3,**LDB2**,LIMA1,**MEIS2**,NIN,NOP56,NPY1R,PAK7,PES1,PRUNE,RCL1,RPS13,SGK3,SLC11A2,SLC20A1,SSTR3,STC2,TNFSF8,TNFSF11,TRHR,TSPAN5	7
Trans-cript	Organ Morphology, Organismal Development, Reproductive System Development and Function	3-beta,17-beta-androstanediol,ABHD5,AR,beta-estradiol,Ca2+,COMP,**DENND5A**,DLG4,DSCAM,**EDN1**,FMOD,FOLH1,HUNK,KCNN2,MED12L,**MEIS2**,MPP1,NFRKB,NLGN3,NLGN4X,**NRXN1**,NXPH1,PITPNM2,POU3F3,Relaxin,S100A2,S100A13,SDK1,**SLC24A2**,SLC24A3,SLC6A9,SRD5A1,SSTR4,SYTL3,ZMIZ1	5
Gene	Gene Expression, Cancer, Developmental Disorder	ABCA6,Actin,AKT1,**ANKH**,**ARSB**,beta-estradiol,C11orf10,C14orf106,C9orf5,**CCDC25**,**CECR1**,CHCHD8,CPA2,CWC25,GINS3,GIP2 (human),HAAO,HNF4A,IFNG,Igtp,**KCNQ3**,**MEIS2**,**MSX1**,**NRXN1**,**PALLD**,PAQR7,**POLR3E**,SH3BGRL2,SRC,SSR2,TMEM43,TMEM87A,TP53,TPRKB,WDFY2	10
	Molecular Transport, Cardiovascular System Development and Function, Cell Morphology	ADAM12,alpha-estradiol,ANXA6,AR,BMX,Ca2+,COMP,Cyclooxygenase,**DENND5A**,**EDN1**,Endothelin,F9,Filamin,GDI1,Girk,K Channel,KCNJ2,MARCKSL1,MME,MXD1,N-acetylglucosamine,Na+,PHLDB2,phosphatidylinositol-3,4,5-triphosphate,PIK3CD,PKN1,PLN,RHO,SCNN1B,SCNN1G,SERPINB2,**SLC24A2**,SNX1,sodium,VSNL1	4
Gene (hESC)	Cardiovascular System Development and Function, Embryonic Development, Organismal Development	**ADAM19**,Akt,**APOA1**,**ARRB1**,**CD34**,Cyclin A,**EIF2AK4**,ERK,ERK1/2,**FGF5**,**FMO1**,**GHR**,Gpcr,GPR174,GPR89A/GPR89B,GPR89C,Histone h3,**ITGA9**,Jnk,**LGR5**,Mapk,NFkB (complex),**NRG1**,**P2RY6**,PDGF BB,PI3K (complex),**PIK3R1**,**PREX1**,**PTPRB**,Rac,**SHANK2**,**SOX6**,**STK10**,TAAR8,VN1R3	18
	Protein Synthesis, Cell Cycle, Gene Expression	2' 5' oas,5430435G22Rik,APLP1,**ASB3**,BPI,Ca2+,**CDS2**,CLEC10A,**CNTN4**,**CNTN5**,COX6A1,CPEB1,**EGFLAM**,Gbp1,**GBP4**,Ifi15,IFNB1,IL6,KIAA0101,KIAA1524,LARGE,**LRRN1**,MX,MYC,NFYB,PITPNM3,**PPP1CC**,PPP1R7,prostaglandin F2alpha,RPL19,RPL27,RPL41,S100A9,**SLCO2A1**,**VTCN1**	10
	Skeletal and Muscular System Development and Function, Renal and Urological Disease, Cell Death	ACSM3,ANKH,ASPN,**ATP2C2**,beta-estradiol,BMP2,BMP8B,C8orf4,Cathepsin,Clec2d (includes others),**CLSTN2**,CNN2,CPXM1,DIP2A,**DNM3**,FASLG,FMO2,**FMO5**,**FMOD**,FXYD5,FYN,GNS,HSD17B6,**KCNJ16**,KDELR,KDELR3,KLK11,MFI2,PDZK1IP1,Pka,**RERG**,**SH3RF2**,SLC13A3,TGFB1,**XYLT1**	9
